# Efficacy of FRO on Acne Vulgaris Pathogenesis

**DOI:** 10.3390/pharmaceutics15071885

**Published:** 2023-07-04

**Authors:** Jung-Eun Kim, Hengmin Han, Yinzhu Xu, Min-Ho Lee, Hyo-Jeong Lee

**Affiliations:** 1 Department of Science in Korean Medicine, College of Korean Medicine, Graduate School, Kyung Hee University, 26, Kyungheedae-ro, Dongdaemun-gu, Seoul 02447, Republic of Korea; kimjulie4717@khu.ac.kr (J.-E.K.); xyz3402@khu.ac.kr (Y.X.); 2 Department of Cancer Preventive Material Development, College of Korean Medicine, Graduate School, Kyung Hee University, 26, Kyungheedae-ro, Dongdamun-gu, Seoul 02447, Republic of Korea; helmin0730@khu.ac.kr; 3 Department of Food Science & Services, Eulji University, Seongnam 13135, Republic of Korea; minho@eulji.ac.kr

**Keywords:** acne vulgaris, *Cutibacterium acnes* (*Propionibacterium acne*), sebum, acne inflammation, *Rhus verniciflua*, *Orostachys japonicus*

## Abstract

Acne vulgaris is a common skin disease characterized by increased sebum production, inflammation, and *Cutibacterium acnes* (CA: formerly *Propionibacterium acnes*) hyperproliferation in pilosebaceous follicles. This study evaluated the efficacy of FRO, a formula composed of fermented *Rhus verniciflua* Stokes and *Orostachys japonicus*, against acne pathogenesis via antimicrobial assessment and an in vitro analysis. Stimulated model cells treated with hormones, CA, or lipopolysaccharide (LPS) were designed based on the characteristics of acne pathogenesis, including inflammation and sebum hypersecretion. High-performance liquid chromatography, disc diffusion, MTS, and western blotting assays were used to examine potential anti-acne effects. FRO was determined to contain phenolics such as gallic acid, fisetin, quercetin, and kaempferol. FRO exerted antimicrobial activity against CA and inhibited reactive oxygen species production that was otherwise increased by LPS or CA in HaCaT cells. Additionally, FRO exerted anti-inflammatory effects by inhibiting iNOS, TNF-α, IL-6, p-STAT-3, and p-NF-κB, which were previously upregulated by LPS or CA in THP-1 and HaCaT cells. FRO inhibited lipogenesis induced by steroid hormones and CA by decreasing FAS and SREBP-1 levels in sebocytes. Additionally, FRO down-regulated the androgen receptor, 5α-reductase, SREBP-1, and FAS levels, which were upregulated by steroid hormone in LNCaP cells. Taken together, our findings suggest that FRO alleviates acne by inhibiting the growth of CA, inflammation, and excess sebum and could be used for functional cosmetics or acne treatments.

## 1. Introduction

FRO is a formula composed of two plant extracts: fermented *Rhus verniciflua* Stokes (FRV) and *Orostachys japonicus* A. Berger (OJ). *Rhus verniciflua* Stokes (RV) [1], a lacquer tree, has been previously studied for its anticancer, antioxidant, antimicrobial, and anti-inflammatory properties [2,3]. Additionally, it has long been used as a traditional medicine and food supplement in South Korea. However, its use has been limited because urushiol, a component of RV, causes allergies. FRV is an urushiol-free RV that has been studied for its antimicrobial, anti-inflammatory, anticancer, and anti-gastritis properties. OJ naturally grows in Korea, China, and Japan and has been used as a traditional medicine to alleviate various symptoms, including fever, inflammation, and bleeding. Additionally, OJ has been shown to possess bioactive, antiproliferative [4,5,6,7,8], anti-angiogenic [9,10], and anti-metastatic [11,12,13] properties against cancer.

Acne vulgaris, a chronic inflammatory disease, is among the top three skin disorders treated by dermatologists and affects more than 80% of young adolescents [14,15]. The main characteristics involved in the pathogenesis of acne include excess sebum production, follicular hyperkeratinization in the sebaceous glands and follicular infundibulum, hyperproliferation of acne-causative microorganisms, and inflammation. Acne can vary in overall severity, which is determined according to the lesion type, size, number, scarring, lesion positions (frequently appearing on the face, back, shoulders, and chest in more severe cases), and post-inflammatory hyperpigmentation [16]. Therefore, acne is a significant health concern with physical and psychosocial effects that may persist long after the treatment of active lesions. Multiple factors affect the pathophysiology of acne, including genetic, hormonal, and environmental factors [17]. Excess sebum production is considered the first step in acne pathogenesis because of the corresponding increase in dihydrotestosterone (DHT) and insulin growth factor-1 (IGF-1) activity [18,19,20,21]. Increased sebum production creates an ideal microenvironment for sustaining the colonization of the causative microorganisms of acne, leading to follicular hyperkeratinization and inflammation [22]. In particular, DHT is produced by the biosynthesis of testosterone by 5α-reductase type 1 in the sebaceous glands of the scalp and skin and plays an essential role in enhancing sebaceous gland cell growth and hyperkeratinization, including micro-comedone production [23]. *Cutibacterium acnes (CA)* is a human skin commensal microorganism. The CA IA1 phylotype is overabundant in the inflammatory lesions of acne patients compared to those of healthy subjects [15,24,25]. CA (phylotype IA1) contributes to the inflammatory process by producing a variety of chemical factors that induce neutrophil chemotaxis and trigger the release of pro-inflammatory cytokines [26,27,28,29]. CA (phylotype IA1) enhances reactive oxygen species (ROS)-associated inflammation by stimulating ROS production by phagocytes and keratinocytes [30,31,32].

Therefore, this study aimed to evaluate the alleviating effect of FRO in in vitro models that were designed to mimic acne pathogenesis.

## 2. Materials and Methods

### 2.1. FRO Manufacturing

The FRO formula used in this study was composed of fermented *Rhus verniciflua* Stokes extract (FRVE) and a 70% ethanol extract of *Orostachys japonicus* A. Berger (OJE); this formula was mixed in a 1:1 ratio by volume. FRVE was purchased from OKKANE (Seoul, South Korea). *O. japonicus*, cultured in 2020, was purchased from Gimcheon Wasong Farming Cooperative (Gimcheon, South Korea). The ground *O. japonicus* was extracted by sonication in 70% ethanol. After extraction, the OJE was filtered through 3M paper. Finally, OJE and FRVE were mixed in a 1:1 ratio by volume and freeze-dried to obtain a powdered form.

### 2.2. High-Performance Liquid Chromatography (HPLC)

FRO was analyzed using an HPLC instrument (Agilent Technologies, Santa Clara, CA, USA) with Hichrome HPLC columns (5 μm, 250 mm  ×  4.6 mm; Hichrome, Theale, UK). The flow rate used was 0.3 mL/min, and the injection volume used was 10 μL. Additionally, 100% methanol was used as the solvent, and a detection wavelength of 254 nm was set. Polyphenol standards (gallic acid, fisetin, quercetin, and kaempferol) were used to characterize the phenolic compounds in the FRO extract. The mobile phase consisted of 0.1% formic acid in water (solvent A) and 100% methanol (solvent B) and was delivered at a flow rate of 0.7 mL/min. The following gradient conditions were used: 0–17 min, 100% B; 17–20 min, 100% B; 20–23 min, 0% B; 23–30 min, 0% B. The detection wavelength used was 254 nm, and the injection volume for all polyphenol samples was 10 μL.

### 2.3. Total Phenolic Content

The total phenolic content (TPC) of the FRO extracts was determined using the Folin-Ciocalteu colorimetric method with some modifications. Various concentrations of gallic acids were used to produce the standard curve. Initially, 25 µL of the FRO extracts were pipetted in triplicate into 96-well plates (Corning Inc., Midland, NC, USA) and incubated for 5 min at room temperature. Furthermore, 25 µL of 10% (*w*/*w*) sodium carbonate was added to basify the mixture. After incubating at room temperature for 1 h, absorbance was determined at 765 nm using a spectrophotometer plate reader (Thermo Fisher Scientific, Waltham, MA, USA). TPC was expressed as mg of gallic acid equivalent (GAE) per gram of fresh weight (mg GAE/g fw) of the sample, using the calibration curve produced by the gallic acid standards (0–100 µg/mL).

### 2.4. Disk Diffusion Susceptibility Testing

*Cutibacterium acnes* [KCTC3314; same as American Type Culture Collection (ATCC) 6919, phylotype IA1] was obtained from the Korea Collection for Type Cultures (KCTC, Daejeon, South Korea). The agar disc diffusion method was used to determine the antimicrobial activity of FRO extract against *C. acnes*. Initially, *C. acnes* (3.12 × 10^7^ CFU) was spread on a BHI agar plate, and 10 millimeter-diameter filter paper disks were placed on the surface of the agar plate. The disks were soaked in FRO (6 mg), and the plates were incubated for 72 h at 37 °C under anaerobic conditions using a gas generation kit (Thermo Fisher Scientific, Waltham, MA, USA). Antimicrobial activity was defined by measuring the diameter of the growth inhibition zone (mm). All experiments were performed in triplicate.

### 2.5. Preparation of Heat-Killed C. acnes

*CA* was cultured anaerobically in brain heart infusion broth (MB cell, Seoul, South Korea) and incubated at 37 °C for 72 h. *C. acnes* was harvested using centrifugation at 4000 rpm for 10 min at 4 °C. The pellet was resuspended in cold Dulbecco’s phosphate buffered saline (Welgene Inc., Daegu, South Korea) and centrifuged three times. Finally, the *C. acnes* pellet was resuspended in Dulbecco’s modified Eagle medium (DMEM) (Welgene Inc., Daegu, South Korea), and the bacterial suspension was heated at 80 °C for 30 min to obtain the heat-killed bacteria. For the stimulation experiment, HaCaT and RAW 264.7 cells were incubated with heat-killed *C. acnes* (5 × 10^7^/mL) in serum-free DMEM for 24 h at 37 °C in 5% CO_2_.

### 2.6. Cell Cultures

A human monocyte cell line, THP-1, was purchased from the Korean Cell Line Bank (KCLB) (Seoul, South Korea) and cultured in RPMI medium containing 10% fetal bovine serum and 1% antibiotics (Welgene Inc., Daegu, South Korea) in a humidified atmosphere at 37 °C and 5% CO_2_. Murine macrophage RAW 264.7 and the human immortalized keratinocyte cell line HaCaT were purchased from KCLB (Seoul, South Korea) and cultured in DMEM containing 10% fetal bovine serum and 1% antibiotics (Welgene Inc., Daegu, South Korea) in a humidified atmosphere at 37 °C and 5% CO_2_. A human immortalized sebocyte cell line, SEB-1, was purchased from Celprogen (Torrance, CA, USA) and cultured in EpiLife Medium (Invitrogen-Gibco, Carlsbad, CA, USA) containing 10% fetal bovine serum, 1% antibiotics (Welgene Inc., Daegu, South Korea), and 5 ng/mL epidermal growth factor in a humidified atmosphere at 37 °C and 5% CO_2_. A human prostate cancer cell line, LNCaP, was purchased from the ATCC. The LNCaP cells were cultured in RPMI-1640 medium supplemented with 10% fetal bovine serum and 1% antibiotics (Welgene Inc., Daegu, Republic of Korea) in a humidified atmosphere at 37 °C and 5% CO_2_.

### 2.7. Cell Viability Assay

The media containing HaCaT and RAW 264.7 cells were seeded into a 96-well plate (SPL Life Science, South Korea) at a density of 1 × 10^4^ cells per well; this plate was then incubated for 24 h. Next, the cells were treated with various concentrations (15.63, 31,25, 62.5, 125, 250, and 500 µg/mL) of FRO and incubated for a further 24 h. The viability of RAW 264.7 and HaCaT cells was evaluated using the CELLOMAX viability kit (Precaregene, Gyeonggi-do, South Korea). CELLOMAX reagent (10 μL) was added to each well and incubated in the dark for 1 h at 37 °C. Optical density was measured at 450 nm using a microplate reader (Tecan, Sunrise, Switzerland). Finally, cell viability was determined as described previously [33].

### 2.8. Nitric Oxide Evalution

NO production was evaluated using the Griess method. RAW 264.7 cells (1 × 10^6^ cells/mL) were seeded in 6-well plates (SPL Life Science, Gyeonggi-do, South Korea) and treated with either 100 or 200 μg/mL FRO. The cells were stimulated with or without LPS (1 μg/mL) and incubated for 24 h. After incubation, 50 µL of each supernatant was collected and transferred to a 96-well microplate (SPL Life Science, Gyeonggi-do, South Korea). Moreover, an equal volume of nitrate solution (100 μM) was added to the supernatant. Sequentially, 50 µL of sulfanilamide solution (Promega, Madison, WI, USA) was added to the wells and kept in the dark for 10 min. After 30 min, NO production was measured at 570 nm using a microplate reader (Tecan, Männedorf, Switzerland).

### 2.9. ROS Assessment

ROS formation was evaluated using the 2′,7′-dichlorofluorescein diacetate (DCF-DA) probe (Abcam, Waltham, MA, USA). HaCaT cells (5 × 10^4^ cells/well) were seeded in 8-well plates and incubated for 24 h until they reached 80% confluence. After incubation, the cells were treated with or without FRO (100 and 200 μg/mL) in phenol-free 10% bovine serum medium for 24 h. Additionally, another group was treated with Tert-butyl hydroperoxide (TBHP) for 4 h to act as a positive control for ROS production. After incubation, the cells were mixed with the DCFDA solution and incubated at 37 °C for 45 min. Fluorescence was monitored at excitation and emission wavelengths of 485 and 530 nm, respectively, using a fluorescence microscope (Nikon, Tokyo, Japan).

### 2.10. Western Blot Analysis

The cells were treated with FRO (0, 100, and 200 µg/mL) and stimulated with lipopolysaccharide (LPS) (1 µg/mL), heat-killed *C. acnes* (5 × 10^7^/µL), or dihydrotestosterone (DHT) (2 nM) for 24 h at 37 °C. Total protein was extracted using RIPA buffer (Thermo Fisher Scientific, Waltham, MA, USA; 1% NP-40, 150 mM NaCl, 50 mM Tris-HCl, pH 7.4, 0.25% sodium deoxycholate, 1 mM Na_3_VO_4_, 1 M EDTA, 1 mM NaF, and a protease inhibitor cocktail). Protein samples were quantified using a Bio-Rad DC Protein Assay Kit II (Bio-Rad, Hercules, CA, USA), separated by electrophoresis on 8%, 10%, or 15% SDS-polyacrylamide gels, and then transferred onto a nitrocellulose blotting membrane (GE Healthcare Life Sciences, Marlborough, MA, USA). The membranes were blocked in 5% skim milk for 1 h, then incubated with the corresponding primary antibodies (Table 1) overnight at 4 °C. After the membranes were exposed to their specific secondary antibodies at room temperature for 2 h, protein levels were assessed using an enhanced chemiluminescence system (GE Healthcare Life Sciences, Marlborough, MA, USA). Finally, ImageJ software (National Institute of Health, Bethesda, MD, USA) was used to quantify each protein band.

### 2.11. Oil Red O Staining

The medium containing SEB-1 sebocytes was seeded in an 8-well plate (SPL Life Science, South Korea) at a density of 5 × 10^3^ cells/well and incubated for 24 h. Furthermore, the cells were treated with FRO (100 µg/mL) and stimulated with heat-killed *C. acnes* (5 × 10^7^/µL) in 2.5% fetal bovine serum medium or DHT (2 nM) in 2.5% fetal calf serum for 24 h at 37 °C and 5% CO_2_. After incubation, Oil Red O staining was performed as previously described [34,35]. The SEB-1 sebocytes were fixed with 4% formalin and stained with 0.6% Oil Red O for 1 h at room temperature. Cell morphology was visualized and photographed using a camera connected to a microscope (Nikon, Tokyo, Japan).

### 2.12. Statistical Analysis

All experiments were performed in triplicate. The data are presented as the mean ± standard deviation [36]. Statistical analyses were conducted using one-way analysis of variance followed by Tukey’s post hoc test using GraphPad Prism 8.0 (GraphPad Software, Boston, MA, USA). Significance was evaluated using the SigmaPlot software. (* *p* < 0.5, ** *p* < 0.01, ** *p* < 0.001).

## 3. Results

### 3.1. FRO Contains Phenolic Compounds

**Figure 1:**
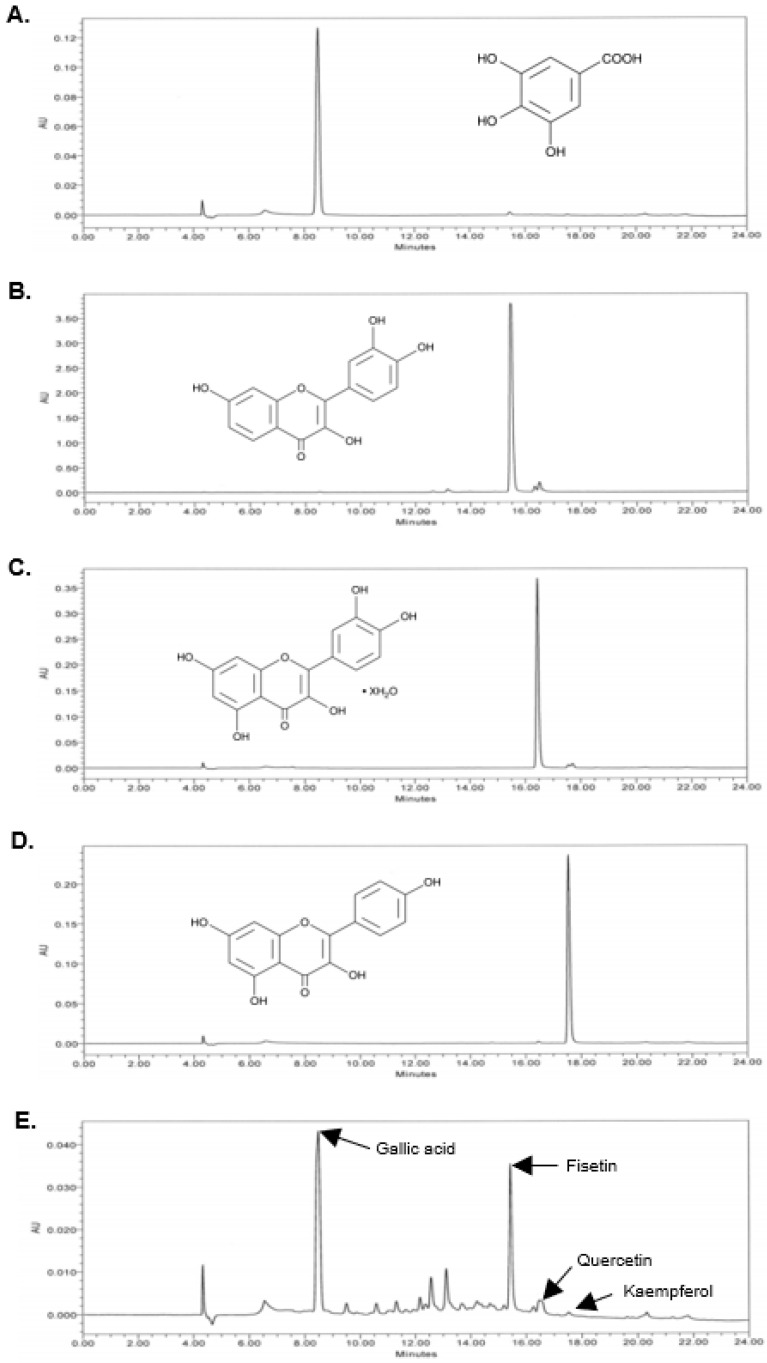
HPLC chromatograms of the FRO extract. Peaks of the four main components: (**A**) gallic acid, (**B**) fisetin, (**C**) quercetin, (**D**) kaempferol, and (**E**) FRO.

The phenolic content in the FRO extract was determined using the total phenolic content (TPC) assay. The FRO extract contained phenolics with a GAE of 118.2 ± 3.16 mg/g (Table 2). We then used HPLC to assess the presence of representative compounds (gallic acid, fisetin, kaempferol, and quercetin) within the two plants composing FRO. As shown in Figure 1, FRO contained gallic acid (PubChem CID 370), fisetin (PubChem CID 5281614), kaempferol (PubChem CID 5280863), and quercetin (PubChem CID 5280343). The corresponding peak indicated that the retention times of gallic acid, fisetin, quercetin, and kaempferol were 8.5, 15.5, 16.5, and 17.6 min, respectively.

### 3.2. FRO Has an Antimicrobial Effect against C. acnes 

**Figure 2:**
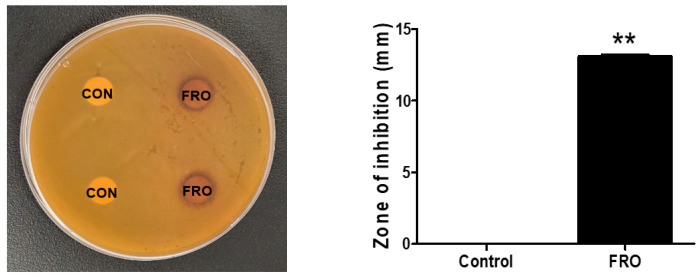
Antimicrobial and antioxidant activity of FRO. Paper disks loaded with DMSO (CON) or FRO. The zone of inhibition is presented as the mean ± SD of three independent experiments. ** *p* < 0.01 versus control groups.

A disk diffusion susceptibility test was utilized to investigate the antimicrobial activity of FRO against CA, a bacterium associated with inflammatory acne. As shown in Figure 2, FRO exhibited antimicrobial activity against CA, producing inhibition zones of 13.1 ± 0.14 mm when 20 μL of FRO 100 mg/mL was loaded into the disk. 

### 3.3. FRO Suppresses CA- and DHT-Induced Lipid Accumulation in SEB-1 Sebocytes 

**Figure 3:**
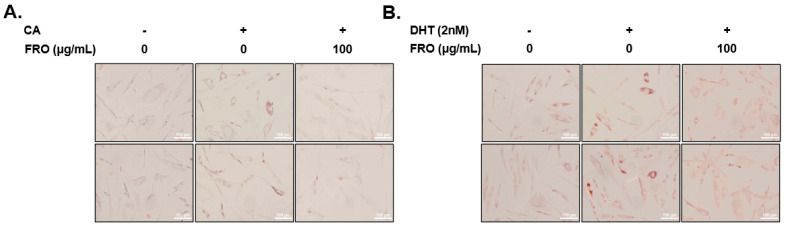

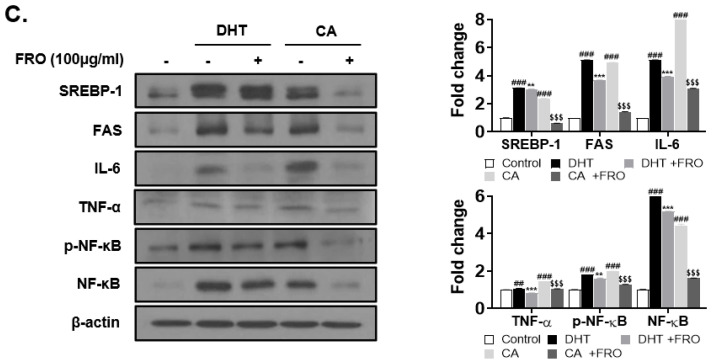
Antilipidemic effect of FRO on CA- or DHT-induced lipid accumulation in SEB-1 cells (**A**) CA- or (**B**) DHT-treated SEB-1 cells were treated with FRO (100 μg) for 24 h. Following FRO treatment, SEB-1 cells were stained with Oil-Red-O dye. (**C**) CA- or DHT-treated cells with FRO-induced sebum production in SEB-1 cells. Cell lysates were subjected to Western blotting to analyze the levels of SREBP-1, FAS, IL-6, TNF-α, p-NF-κB, NF-κB, and β-actin. The results are presented as the means ± SD of three independent experiments. ^##^ *p* < 0.01, ^###^ *p* < 0.001 (control versus DHT- or CA-induced control), ** *p* < 0.01 and *** *p* < 0.001 versus the DHT-induced control group, and ^$$$^ *p* < 0.001 versus the CA-induced control group.

Sebum production is considered the first step in acne pathology; this accumulation aggravates acne by initiating hyperkeratinization and CA colonization in the sebaceous glands. Following treatment with CA or DHT, increased lipid accumulation within SEB-1 cells was observed via Oil-red-O staining. CA- or DHT-stimulated SEB-1 cells with FRO treatment exhibited increased or decreased lipid accumulation, respectively (Figure 3A,B). In addition, the corresponding effects of CA, DHT, and FRO on lipid accumulation were verified by observing the resulting changes in cellular lipogenic protein levels with Western blotting. As shown in Figure 3C, DHT and CA treatment increased SREBP-1 and FAS protein levels. Specifically, a three- and two-fold increase in SREBP-1 protein levels was observed following DHT and CA treatment, respectively, compared to the no-treatment group. FAS levels in the DHT- and CA-treated groups were fourfold higher compared with those of the no-treatment group. FRO treatment significantly reduced SREBP-1 and FAS levels in SEB-1 cells that were otherwise increased by CA and DHT treatment. Overall, FRO reduced the CA-mediated increase in levels of SREBP-1 and FAS to a greater extent than the DHT-mediated increase in levels of these proteins. CA and DHT also significantly upregulated inflammatory markers (IL-6 and TNF-α). Nonetheless, CA treatment resulted in higher IL-6 and TNF-α expression levels than DHT treatment. Nonetheless, FRO inhibited this increase in cytokine levels. Furthermore, NF-κB, a transcription factor that regulates inflammatory cytokines, was upregulated following DHT and CA treatment of SEB-1 cells. FRO similarly suppressed the levels of both NF-κB and phosphorylated NF-κB. 

### 3.4. FRO Has an Anti-Androgenic and Lipogenic Effect on DHT-Induced LNCaP Cells 

**Figure 4:**
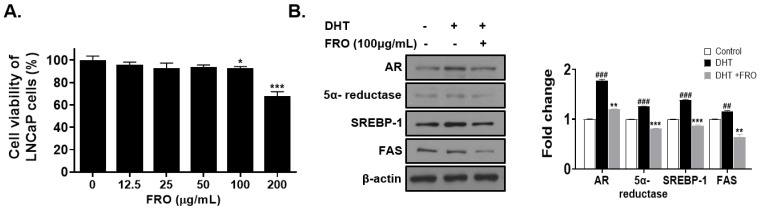
Anti-androgenic and anti-lipogenic effects of FRO on DHT-treated LNCaP cells (**A**) LNCaP cells were treated with the indicated concentrations of FRO for 24 h (*n* = 3). An MTS assay was used to measure cell viability. The results are presented as the mean ± SD of three independent experiments. * *p* < 0.05 and *** *p* < 0.001 versus control groups. (**B**) DHT-treated SEB-1 cells were treated with FRO (100 μg) for 24 h. Cell lysates were subjected to Western blotting to analyze the levels of AR, 5α- reductase, SREBP-1, FAS, and β-actin. The results are presented as the mean ± SD of three independent experiments. ^##^ *p* < 0.01, ^###^ *p* < 0.001 (control versus DHT-induced control), ** *p* < 0.01, and *** *p* < 0.001 versus the DHT-induced control group.

Sebum production is regulated by androgen hormones such as DHT, which is converted from testosterone by 5-α-reductase. LNCaP cells are androgen-dependent prostate cancer cell lines that are used as models to evaluate anti-androgenic effects. Additionally, DHT has been previously used to induce androgenic and lipogenic activity. Initially, an MTS assay was used to determine the effect of FRO concentration on LNCaP cell viability (Figure 4A). Except for 200 μg/mL FRO, the different concentrations of FRO resulted in cell viability of 95–92% (Figure 4A). DHT stimulated androgen receptor (AR), 5α-reductase, SREBP-1, and FAS levels. However, FRO treatment reduced the levels of AR, 5α-reductase, SREBP-1, and FAS (Figure 4B).

### 3.5. FRO Inhibits Inflammation-Associated Reactive Oxygen Species (ROS) and Nitric Oxides (NO)

**Figure 5:**
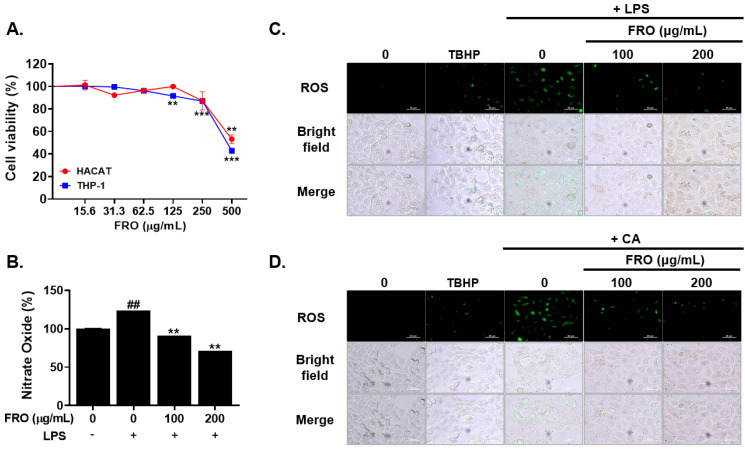
Inhibitory effect of FRO on ROS and NO production in LPS-or CA-treated THP-1 or HaCaT cells. (**A**) HaCaT and THP-1 cells were treated with the indicated concentrations of FRO for 24 h (*n* = 3). An MTS assay was used to measure cell viability. The results are presented as the mean ± SD of three independent experiments. ** *p* < 0.01 and *** *p* < 0.001 versus control groups. (**B**) THP-1 cells were treated with FRO (0, 100, and 200 μg/mL) for 24 h (*n* = 3). Nitric oxide (NO) production was evaluated using the Griess method. The results are presented as the mean ± SD of three independent experiments. ^##^ *p* < 0.01 (control versus the LPS-treated control group) and ** *p* < 0.05 (versus the LPS-treated control group). (**C**) LPS- or (**D**) CA-treated HaCaT cells were treated with FRO (0, 100, and 200 μg/mL) for 24 h or TBHP 50 μM (positive control) for 4 h. The HaCaT cells were stained with a DCFDA solution and incubated at 37 °C for 45 min. Fluorescence was monitored using a fluorescence microplate reader.

NO and ROS can activate inflammatory responses. In the present study, LPS was observed to increase NO secretion in THP-1 cells. The LPS-dependent increase in NO secretion was 1.23-fold greater than that of the control. Nonetheless, 100 and 200 μg/mL FRO suppressed this increase in NO levels by up to 29% without affecting cell viability (Figure 5A,B). LPS and CA enhanced ROS production in HaCaT cells. This enhanced ROS production was reduced by FRO treatment (Figure 5C,D).

### 3.6. FRO Confers an Anti-Inflammatory Effect by Regulating Two Transcription Factors, STAT-3 and NF-κB 

**Figure 6:**
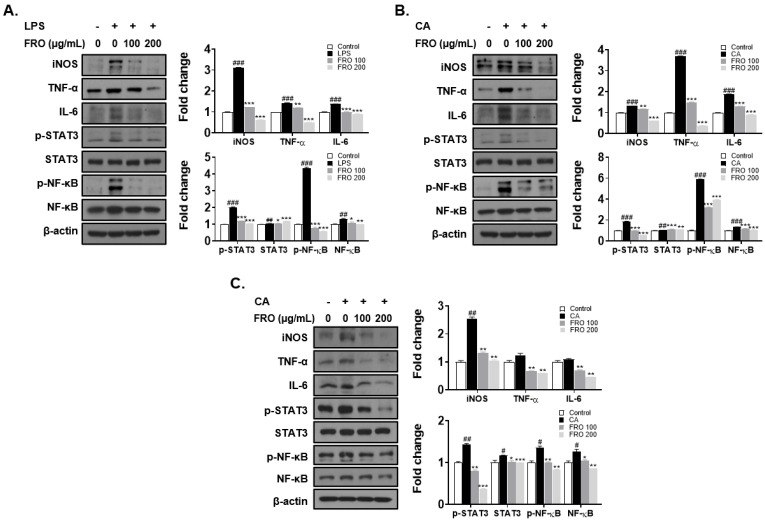
Anti-inflammatory effect of FRO. (**A**) LPS- or (**B**) CA-treated THP-1 cells and (**C**) CA-treated HaCaT cells were treated with FRO for 24 h (*n* = 3). Cell lysates were subjected to Western blotting to analyze the levels of iNOS, TNF-α, IL-6, p-STAT3, STAT3, p-NF-κB, NF-κB, and β-actin. The results are presented as the mean ± SD of three independent experiments. ^#^ *p* < 0.05, ^##^ *p* < 0.01, ^###^ *p* < 0.001 (control versus LPS- or CA-induced control group) * *p* < 0.05, ** *p* < 0.01, and *** *p* < 0.001 versus the LPS- or CA-induced control group).

To investigate the anti-inflammatory effect of FRO, THP-1 cells and HaCaT cells were treated with LPS or CA to establish inflammatory models. LPS significantly increased the levels of inflammatory factors (iNOS, TNF-α, and IL-6) in THP-1 cells (Figure 6A). CA, similar to LPS, enhanced the levels of inflammatory factors in THP-1 cells (Figure 6B) and HaCaT cells (Figure 6C). Overall, FRO reduced the LPS- or CA-induced increase in iNOS, TNF-α, and IL-6 levels in THP-1 and HaCaT cells (Figure 6A–C). Furthermore, NF-κB and STAT-3, transcription factors that regulate the levels of inflammatory response cytokines, were upregulated in THP-1 and HaCaT cells following LPS and CA treatment. Nonetheless, FRO suppressed the increased levels of NF-κB, phosphorylated NF-κB, and phosphorylated STAT-3.

## 4. Discussion

Acne vulgaris is a common skin disease that may be of significant physical and psychosocial concern depending on the severity and duration of treatment. Multiple genetic, hormonal, and environmental factors lead to the development of acne vulgaris. Acne pathogenesis involves significantly increased sebum production, inflammation, and *C. acnes* (formerly *Propionibacterium acnes*) hyperproliferation. *C. acnes* is associated with the inflammatory response and lipogenesis within the pilosebaceous unit [27,37].

*C. acnes* contributes to the augmentation of sebaceous lipogenesis by increasing 15-deoxy-12,14-prostaglandin J2 production, leading to enhanced production of diacylglycerol acyltransferase 1-dependent triacylglycerol [38]. Further, the androgen DHT enhances cell growth of the sebaceous gland and sebaceous lipogenesis [39,40,41,42]. SREBP-1 acts as a transcription factor to regulate several lipogenic genes, including FAS, ACC, and SCD-1, which are involved in fatty acid biosynthesis [43].

*C. acnes* initiates the inflammatory process of acne pathogenesis by triggering the production of ROS and inflammatory cytokines via Toll-like receptor 2, peroxisome proliferator-activated receptor, the mTOR pathway, and the innate immune system [44,45,46,47].

The molecular mechanisms underlying oxidative stress and inflammation-induced cell injury in acne have been previously investigated. TLR-mediated NF-κB and STAT signaling have been determined to be key acne mechanisms. 

In this study, we evaluated the anti-bacterial, antioxidant, anti-lipogenic, and anti-inflammatory properties of FRO in CA-, LPS-, and DHT-stimulated acne cell models that were based on acne pathogenesis. The corresponding results indicated that FRO suppressed CA growth and sebum production by the sebaceous glands, which was otherwise induced by hormones or causative bacteria; specifically, FRO inhibited the lipogenesis-related proteins SREBP-1 and FAS caused by CA-or DHT-induced sebum production in SEB-1 cells. Additionally, FRO conferred anti-inflammatory effects, leading to a reduction in cytokines, ROS, and NO production via inhibition of the NF-κB/STAT-3 pathway; this alleviated LPS- and CA-stimulated inflammation and oxidative stress in THP-1, RAW 264.7 (Supplementary Data), HaCaT, and SEB-1 cells.

In the present study, the TPC of FRO was determined to be 118.2 mg GAE/g (Table 1). FRV and *Orostachys japonicus* A. Burger [48], two ingredients of FRO, contain gallic acid, fisetin, quercetin, and kaempferol, alongside many phenolics, such as protocatechuic acid, fustin, sulfuretin, butein, hexacosanol, 4-hydroxybenzoic acid, 3,4-dihydroxybenzoic acid, and methyl gallate [34,49,50,51]. The HPLC peaks of FRO that were not identified with the standards used in this study may be these phenolics (Figure 1). Nonetheless, further studies are needed to characterize the FRO compounds and verify their effect on acne.

Our results demonstrated that FRO exerts anti-inflammatory effects by inhibiting the LPS- and CA-induced increase in cytokines and ROS production and regulating the transcription factors involved in the expression of inflammatory factors. Gallic acid, fisetin, quercetin, and kaempferol, which are components of FRO (Figure 1), possess anti-inflammatory and antioxidant effects. These components have been studied for their anti-inflammatory effects and mechanisms of action in vitro and in vivo. Specifically, these components exert anti-inflammatory effects by suppressing the release of inflammatory cytokines, such as IL-6, IL-1β, and TNF-α, and TLR-mediated NF-κB, STAT, or MAPK signaling [52,53,54,55]. Moreover, gallic acid, quercetin, and kaempferol have been previously reported to possess anti-acne vulgaris effects [56,57,58,59,60,61,62,63]. These reports indicate that gallic acid, fisetin, quercetin, and kaempferol may be closely associated with the favorable anti-acne effects of FRO.

FRO has been investigated in clinical trials for cosmetic applications [64]. In this prior study, 26 subjects were randomly assigned to toner and lotion containing 10% FRO or placebo toner and lotion; individuals then used the provided cosmetics twice a day for 6 weeks. The facial skin’s moisture, elasticity, and pores were assessed at 0, 2, 4, and 6 weeks after treatment began. Facial moisture and elasticity levels increased significantly in the FRO-treated group after 6 weeks. Furthermore, pore size decreased significantly in the FRO group compared to those in the placebo group 2 weeks after treatment began. This report has limitations owing to the lack of acne evaluation; nonetheless, it demonstrated that FRO has potential in cosmetics application.

## 5. Conclusions

There are several acne medications currently available, which primarily include combinations of oral and topical anti-microbials and retinoid agents, alongside alternative options such as laser/light therapy, chemical peeling, and hormonal agents. However, these treatment strategies have side effects and are relatively expensive. Natural products with nontoxicity, multi-targeting potential, and high efficacy may ameliorate acne. The present study provided evidence that FRO exerts potent anti-acne effects. FRO can ameliorate acne, which has been attributed to its antimicrobial, anti-inflammatory, anti-lipogenic, and antioxidant effects in CA-treated SEB-1, THP-1, RAW 264.7 cells (Supplementary Data), and HaCaT cells. FRO treatment of acne vulgaris is predominantly associated with its exceptional anti-sebum production ability via the regulation of SREBP-1 and modulation of inflammation and oxidants via the NF-κB and STAT signaling pathways. In conclusion, FRO can be used as a functional cosmetic or acne treatment.

## Figures and Tables

**Table 1:** Antibodies used in this study.

Antibody	Company	Dilution	Product No.
p-STAT-3	Cell Signaling	1:2000	9145
STAT-3	Cell Signaling	1:2000	12640
p-NF-κB	Cell Signaling	1:1000	3031
NF-κB	Cell Signaling	1:1000	8242
TNF-α	Cell Signaling	1:1000	3707
IL-6	Cell Signaling	1:1000	12912
SREBP-1	Santa Cruz	1:1000	sc-365513
FAS	Cell Signaling	1:5000	3180
AR	Cell Signaling	1:1000	3202
iNOS	Bioss	1:1000	bs-2072
β-actin	Sigma-Aldrich	1:20,000	A5316
goat anti-rabbit IgG (HRP)	Abcam	1:5000	ab97051
goat anti-mouse IgG (HRP)	Jackson	1:5000	115-035-003

**Table 2:** Total phenolic content (TPC) of FRO extract.

TPC	FRO
mg GAE/g	118.2 ± 3.16

## Data Availability

Not applicable.

## Supplementary Materials

The following supporting information can be downloaded at: https://www.mdpi.com/article/10.3390/pharmaceutics15071885/s1. Figure S1. Inhibitory effect of FRO on NO production in LPS-treated Raw 264.7 cells; Figure S2. Anti-inflammatory effect of FRO in LPS- or CA-treated RAW 264.7 cells.
