# Supplementary material for: Efficacy of FRO on Acne Vulgaris Pathogenesis
Source: Pharmaceutics. 2023 Jul 4;15(7):1885. doi: 10.3390/pharmaceutics15071885 (PMC10384752; doi:10.3390/pharmaceutics15071885)
Supplement: Supplementary file 1 [file pharmaceutics-15-01885-s001.zip › pharmaceutics-2436843-supplementary.pdf]

pharmaceutics

MDPI

Supplementary Materials: Efficacy of FRO on acne vulgaris pathogenesis

Jung-Eun Kim, Hengmin Han, Yinzhu Xu, Min-Ho Lee and Hyo-Jeong Lee

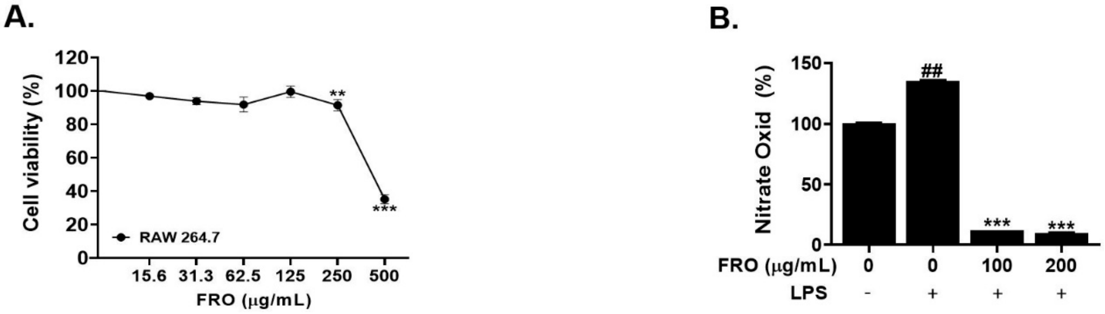

Figure S1. Inhibitory effect of FRO on NO production in LPS-treated Raw 264.7 cells. (A) RAW 264.7 cells were treated with indicated concentrations of FRO for 24 h (n = 3). MTS assay was used to measure cell viability. The results are presented as the mean ± SD of three independent experiments; $^{**}p < 0.01$ and $^{***}p < 0.001$ versus control groups. (B) RAW 264.7 cells were treated with FRO (0, 100, and 200 μg/mL) for 24 h (n = 3). The nitric oxide (NO) production was evaluated using the Griess method. The results are presented as the mean ± SD of three independent experiments; $^{\#\#}p < 0.01$ (control versus LPS- treated control group) and $^{***}p < 0.001$ versus the LPS-treated control group.

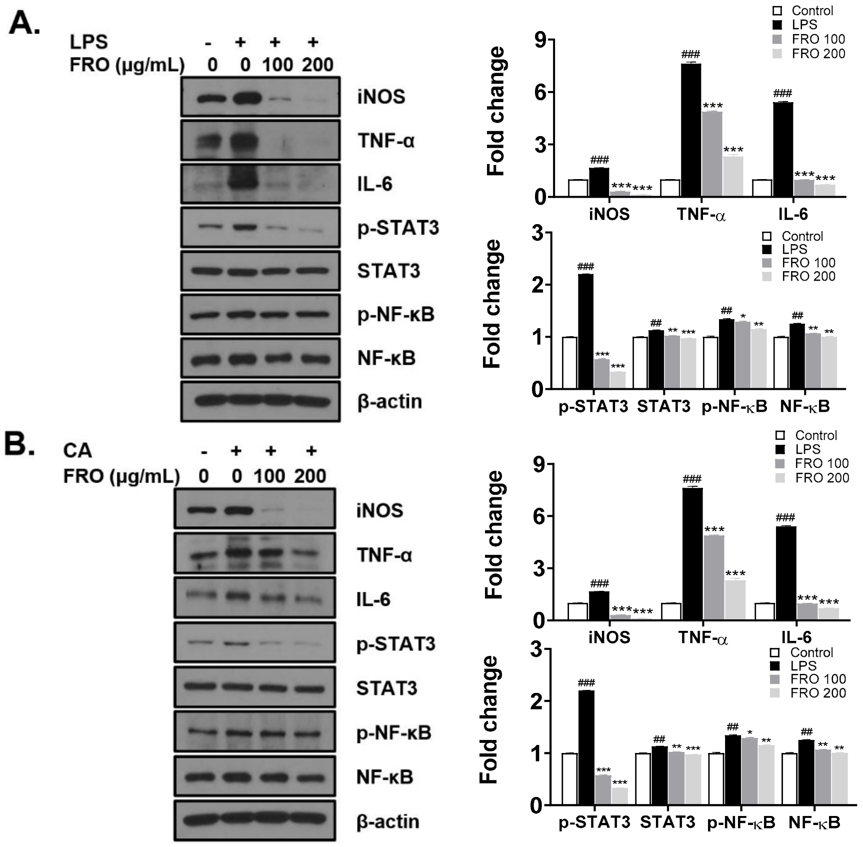

Figure S2. Anti-inflammatory effect of FRO in (**A**) LPS- or (**B**) CA-treated RAW 264.7 cells. The cells were treated with FRO for 24h (n = 3). Cell lysates were subjected to western blotting to analyze the expression of iNOS, TNF-α, IL-6, p-STAT3, STAT3, p-NF-κB, NF-κB, and β-actin. The results are presented as the mean ± SD of three independent experiments. $^{\#\#}p < 0.01$, $^{\#\#\#}p < 0.001$ (control vs. LPS-

or CA- induced control group), *$p < 0.05$, **$p < 0.01$, and ***$p < 0.001$ versus the LPS- or CA- induced control group.
